# Long-term safety and effectiveness of paroxysmal atrial fibrillation ablation using a porous tip contact force-sensing catheter from the SMART SF trial

**DOI:** 10.1007/s10840-020-00780-4

**Published:** 2020-05-27

**Authors:** Andrea Natale, George Monir, Anshul M. Patel, Robert S. Fishel, Francis E. Marchlinski, M. Craig Delaughter, Charles A. Athill, Daniel P. Melby, Mario D. Gonzalez, Ramesh Hariharan, Brett Gidney, Tiffany Tan, Larry A. Chinitz

**Affiliations:** 1 Texas Cardiac Arrhythmia Research Foundation, Austin, TX USA; 2 Florida Hospital Cardiovascular Research, Orlando, FL USA; 3 Emory St Joseph’s Hospital, Atlanta, GA USA; 4 Florida Electrophysiology Associates, Atlantis, FL USA; 5 University of Pennsylvania, Philadelphia, PA USA; 6 HeartPlace, Bedford, TX USA; 7 San Diego Cardiac Center, San Diego, CA USA; 8 Abbott Northwestern Hospital, Minneapolis, MN USA; 9 Penn-State Milton S. Hershey Medical Center, Hershey, PA USA; 10 UT Physicians - EP Heart, The Woodlands, TX USA; 11 Santa Barbara Cottage Hospital, Santa Barbara, CA USA; 12 Biosense Webster, Inc., Irvine, CA USA; 13 NYU Langone Medical Center New York University, New York, NY USA

**Keywords:** Efficacy, Contact force, Paroxysmal atrial fibrillation, Porous tip, Radiofrequency catheter ablation, SmartTouch SF

## Abstract

**Purpose:**

The prospective, multicenter SMART SF trial demonstrated the acute safety and effectiveness of the 56-hole porous tip irrigated contact force (CF) catheter for drug-refractory paroxysmal atrial fibrillation (PAF) ablation with a low primary adverse event rate (2.5%), leading to FDA approval of the catheter. Here, we are reporting the long-term effectiveness and safety results that have not yet been reported.

**Methods:**

Ablations were performed using the 56-hole porous tip irrigated CF catheter guided by the 3D mapping system stability module. The primary effectiveness endpoint was freedom from atrial tachyarrhythmia **(**including atrial fibrillation, atrial tachycardia, and/or atrial flutter), based on electrocardiographic data at 12 months. Atrial tachyarrhythmia recurrence occurring 3 months post procedure, acute procedural failures such as lack of entrance block confirmation of all PVs, and undergoing repeat procedure for atrial fibrillation in the evaluation period (91 to 365 days post the initial ablation procedure) were considered to be effectiveness failures.

**Results:**

Seventy-eight patients (age 64.8 ± 9.7 years; male 52.6%; Caucasian 96.2%) participated in the 12-month effectiveness evaluation. Mean follow-up time was 373.5 ± 45.4 days. The Kaplan-Meier estimate of freedom from 12-month atrial tachyarrhythmia was 74.9%. Two procedure-related pericardial effusion events were reported at 92 and 180 days post procedure. There were no pulmonary vein stenosis complications or deaths reported through the 12-month follow-up period.

**Conclusions:**

The SMART SF 12-month follow-up evaluation corroborates the early safety and effectiveness success previously reported for PAF ablation with STSF.

## Introduction

Atrial fibrillation (AF) is the most common arrhythmia diagnosed in clinical practice and the prevalence is increasing as the US population ages [1, 2]. Without treatment, AF is associated with significant morbidity and mortality, including stroke, embolism, and heart failure [3–5]. In patients with paroxysmal atrial fibrillation (PAF) refractory to drug treatment, radiofrequency (RF) catheter ablation is an important treatment option that has been shown to be superior to antiarrhythmic drug treatment [6, 7].

The THERMOCOOL SMARTTOUCH® SF Catheter (STSF), a recently developed contact force (CF) catheter, enables real-time sensing of the catheter-to-tissue contact during RF ablation [8]. A 56-hole porous tip has been incorporated in the original CF-sensing catheter design to produce more uniform cooling with less fluid delivery [9, 10]. Compared with the traditional six-hole irrigation system, the porous tip catheter improves procedural efficiency and reduces fluid delivery [11, 12], while maintaining the safety profile for PAF ablation [9].

The acute data from the SMART SF study showed the STSF catheter to be safe with high early effectiveness [11]. A low primary adverse event (AE) rate of 2.5% (4/159) was observed, which is comparable with complication rates for traditional irrigated catheters [7, 8]. There were no unexpected serious AEs related to the study device. Acute effectiveness, defined as confirmation of entrance block for all targeted pulmonary veins (PVs), was 96.2% for subjects who undergone RF ablation. Fluid delivered via the STSF catheter was 44.7% lower than previously reported with the traditional 6-hole non-CF irrigated THERMOCOOL® catheter [7] and 52.2% lower than with the 6-hole irrigated CF THERMOCOOL SMARTTOUCH® catheter [8, 11].

To obtain data on long-term performance, a 12-month extension of the SMART SF study across multiple centers was conducted.

## Methods

### Study design

The SMART SF study (NCT 02359890) design was previously described. Briefly, it was a prospective, multicenter, non-randomized clinical evaluation of THERMOCOOL SMARTTOUCH® SF Catheter (STSF) for drug-refractory PAF ablation. Patient eligibility criteria in this predicate safety study included age ≥ 18 years, symptomatic PAF with at least one documented AF episode within 1 year prior to enrollment, and a physician’s note indicating a diagnosis of recurrent, self-terminating AF. Eligible patients had also previously failed at least one class I or III antiarrhythmic drug (AAD) or AV nodal blocking agent, or were intolerant to an AAD. The primary safety endpoint was met and has been previously reported [11]. The effectiveness population (EP) included patients who met all inclusion and exclusion criteria and re-consented to participate in the effectiveness phase.

The effectiveness phase of the trial, described herein, was an extension of the original safety phase that was added to evaluate 12-month effectiveness for PAF patients treated with the study catheter. The follow-up period was extended through 12 months after the initial procedure for subjects who could be re-consented. The effectiveness phase was approved by the institutional review boards or ethics committees at all participating centers and was conducted in accordance with the International Conference on Harmonization (ICH) Harmonized Tripartite Guidelines for Good Clinical Practice. To be included in the effectiveness evaluation, subjects were required to have participated in the safety phase, had ablation procedures for PAF performed with the STSF catheter, and provided consent for the extended follow-up.

The 12-month effectiveness endpoint was defined as freedom from documented AF, atrial tachycardia (AT), and/or atrial flutter (AFL)—hereinafter collectively referred to as atrial tachyarrhythmia—based on electrocardiographic data. This included ECG, telemetry strip, and 48-h Holter monitor collected at the 6-to-9- and 12-month visits during the evaluation period (91–365 days after the initial procedure). In addition, 12-month effectiveness success required freedom from acute procedural failures, which were defined as inability to confirm entrance block of all PVs at the end of ablation procedure or a use of non-study catheter for the treatment of study arrhythmia for the initial procedure. Undergoing repeat ablation for atrial tachyarrhythmia was considered an effectiveness failure.

In the effectiveness phase, all adverse events (AEs) that occurred from 30 days post-ablation through the 12-month follow-up visit were collected and reported. Procedure- or device-related serious AEs (SAEs) occurring within 30 days of the procedures were already reported in the publication of acute data [11]. For each reported AE, the severity, clinical outcome, and causality were monitored until adequately resolved or explained.

### Statistical analysis

Descriptive statistics of demographics and baseline characteristics were calculated for patients in the EP, as well as for the original enrolled population and the subjects that did not re-consent. The 12-month effectiveness success rate was calculated by dividing the number of subjects who were free from atrial tachyarrhythmia recurrence or other effectiveness failures by the total number of subjects in the EP. The breakdown of reasons for effectiveness failures was also summarized. The success rate was compared with the performance goal of 50% using the exact test for a binomial proportion. The sample size of 78 in the EP provided at least 80% power for this test, with a one-sided significance level of 0.05.

The Kaplan-Meier analysis with two-sided 90% confidence intervals (CI) was used to estimate the time to first atrial tachyarrhythmia recurrence. To evaluate the association between demographics, baseline medical history, and procedural data with the 12-month effectiveness endpoint, univariable and multivariable models were fit to the data. If any statistically significant associations were observed at a 0.10 level, the variables were considered for the multivariable model. Multivariate logistic regression models were then used to explore the associations between key patient or procedural characteristics and the 12-month effectiveness endpoints. These models were used to estimate odds ratios (ORs) and 95% CIs for gender, duration of AF history, previous use of class I AAD, baseline LA diameter, average contact force during ablation procedure, and the use of anticoagulation and cardiovascular medications 90 days post index procedure.

## Results

### Baseline characteristics and demographics

**Fig. 1:**
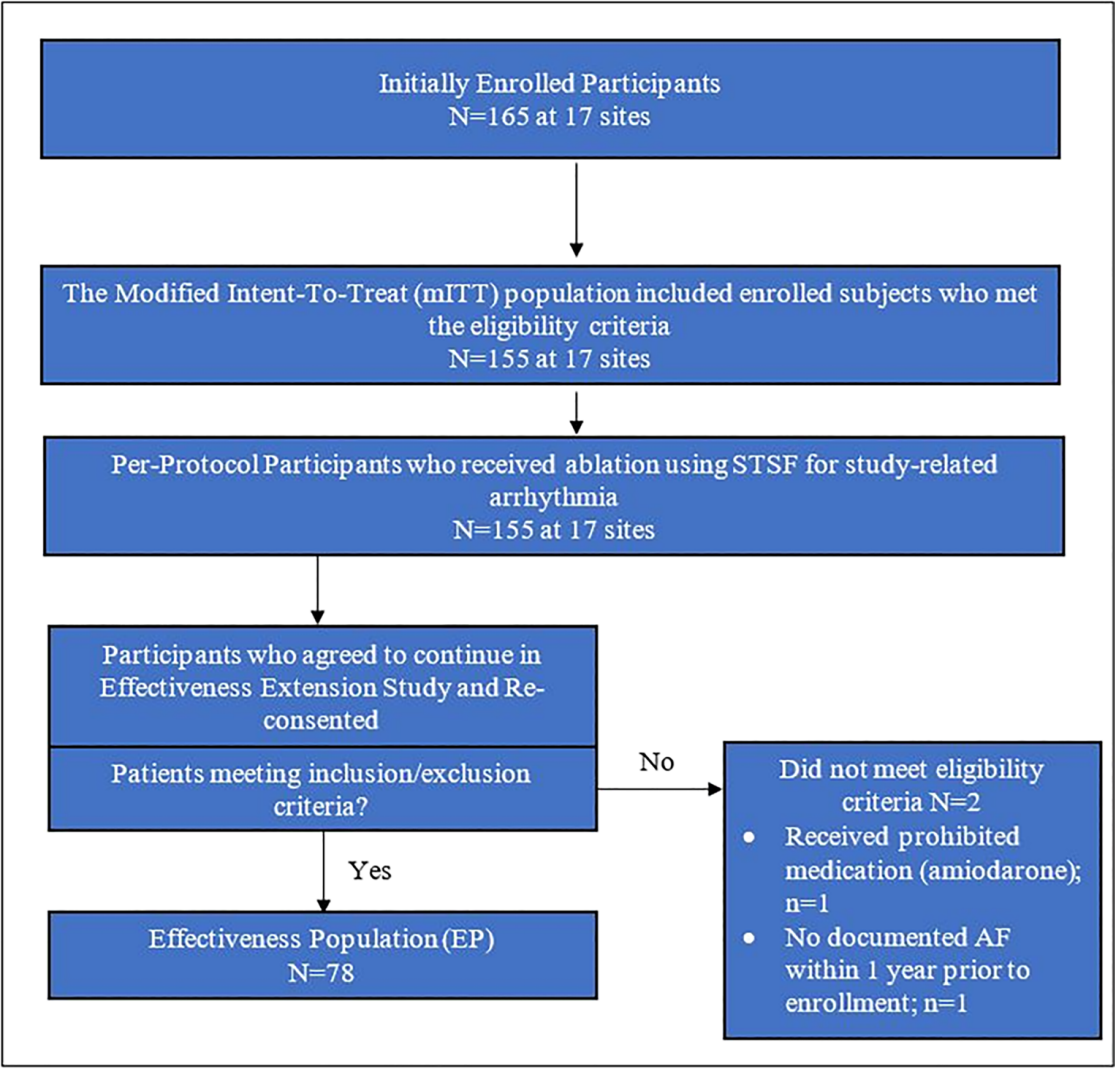
Patient disposition

Eighty (80) of 135 subjects (from sites who agreed to participate in the effectiveness phase) from 13 centers re-consented to participate in the 12-month effectiveness evaluation, of which 2 were excluded because they did not meet eligibility criteria. Thus, a total of 78 patients were included in the EP. Figure 1 displays the details of the patient disposition flow chart.

Table 1 summarizes the demographics and pre-existing baseline medical conditions for the original SMART SF trial and for the subset of patients in the EP. Overall, the EP was similar to the full patient population. In the EP, the majority of participants were white (96.2%) and male (52.6%), with a median age of 64.8 ± 9.7 years. The most common pre-existing medical conditions in the EP were hypertension (61.5%), atrial flutter (30.8%), and coronary artery disease (20.5%). All enrolled patients and patients in the EP experienced symptomatic AF for an average of 47.9 and 41.3 months prior to enrollment, respectively. The median duration from patients’ initial AF diagnosis to enrollment was 24.0 months (all enrolled) and 19.5 months (EP). The mean follow-up duration for the 78 patients was 373.5 ± 45.38 days from index procedure. Compared with those who elected not to be included in the study extension through 12 months of follow-up (non-EP), patients in the EP (*n* = 78) had a slightly higher proportion of female participants (47.4% vs. 32.5%), higher mean age (64.8 vs. 60.5 years), higher proportion of patients with coronary artery disease (20.5% vs. 15.6%), and more symptomatic AF at baseline.

**Table 1:** Baseline demographic and patient characteristics

Characteristics	Enrolled *n* = 165*	EP *n* = 78	Non-EP *n* = 77
Age, mean ± SD, years	62.7 ± 10.4	64.8 ± 9.7	60.5 ± 10.2
Male	95 (57.9)	41 (52.6)	52 (67.5)
Race, Caucasian	159 (97.0)	75 (96.2)	76 (98.7)
Pre-existing baseline medical condition(s)*			
Coronary artery disease	29 (17.8)	16 (20.5)	12 (15.6)
Congestive heart failure	6 (3.7)	1 (1.3)	4 (5.2)
Myocardial infarction	6 (3.7)	1 (1.3)	5 (6.5)
Significant valve disease	3 (1.8)	2 (2.6)	1 (1.3)
Thromboembolic event	10 (6.1)	7 (9.0)	3 (3.9)
Atrial flutter	51 (31.3)	24 (30.8)	23 (29.9)
Diabetes	23 (14.1)	12 (15.4)	10 (13.0)
Hypertension	93 (57.1)	48 (61.5)	42 (54.5)
Baseline transthoracic echocardiogram (TTE)			
Mean left ventricle ejection fraction (%)	60.1	59.0	60.8
Mean LA diameter from PLAX (mm)	38.8	38.4	39.3
AF history			
Mean AF duration, months prior to enrollment	47.9	41.3	50.8
Median AF duration, months prior to enrollment	24.0	19.5	24.0
AF symptoms			
Chest pain	16 (9.8)	10 (12.8)	6 (7.79)
Dizziness	46 (28.2)	23 (29.5)	21 (27.27)
Dyspnea	69 (42.3)	34 (43.6)	35 (45.45)
Palpitations	129 (79.1)	58 (74.4)	64 (83.12)
Syncope	9 (5.5)	5 (6.4)	3 (3.90)
Weakness	30 (18.4)	21 (26.9)	9 (11.69)
Nausea	9 (5.5)	7 (9.0)	2 (2.60)
Lightheadedness	46 (28.2)	27 (34.6)	19 (24.68)
Fatigue	84 (51.5)	41 (52.6)	38 (49.35)

Values are *n* (%) unless specified

*EP*, effectiveness population; *SD*, standard deviation; *PLAX*, parasternal long axis

*Pre-existing baseline medical conditions, AF history, and AF symptoms were summarized based on 163 enrolled subjects who have available medical history and AF baseline data

During the effectiveness phase of the study, the baseline medical history case report form was unlocked for patients enrolled in this portion of the study, which resulted in including the identification of other arrhythmias such as premature ventricular contractions and atrial tachycardia

### Effectiveness

**Fig. 2:**
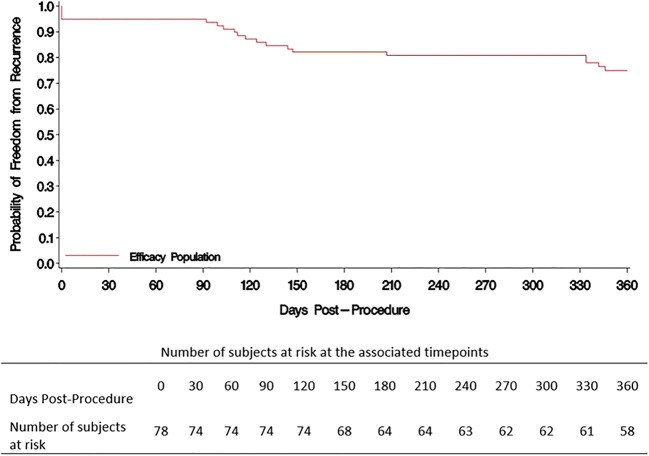
Kaplan-Meier freedom from tachyarrhythmia (effectiveness population, *N* = 78)

The Kaplan-Meier estimate of freedom from atrial tachyarrhythmia recurrence 12 months was 74.9% (90% CI [65.0%, 84.7%]) with 12 subjects experiencing recurrence of atrial fibrillation. The lower bound of the 90% confidence interval was greater than the pre-determined performance goal of 50%; thus, the effectiveness endpoint was met (Fig. 2). Table 2 summarizes the reasons for the 19 patients who failed the 12-month effectiveness criteria.

**Table 2:** 12-month effectiveness (effectiveness population, *N* = 78)

Primary outcome and reasons for failure	*n* (%)
Total successes	59/78 (75.6%)
Total failures	19/78 (24.4%)
AF/AT/AFL recurrence*	12 (15.4%)
AT	2 (2.6%)
AF	10 (12.8%)
Acute failure**	4 (5.1%)
Repeat ablation post-blanking period	3 (3.8%)

*AF/AT/AFL = atrial fibrillation/atrial tachycardia/atrial flutter

**Acute failure is defined as lack of entrance block confirmation for all PV

Using a univariate model, gender, duration of AF history, previous class I AAD history, baseline LA dimension, average contact force, and post-AF cardiac medications were identified as predictors of failure in regard to 12-month failure (Table 3). Using a multivariate logistic regression model, class I AAD history was a significant predictor of failure at *p* ≤ 0.05 (OR 5.3, 95% CI [1.32, 21.55]). Females tended to have lower 12-month success rate compared with males with OR = 3 (95% CI 0.79, 11.27). An additional month in duration of AF history increased the odds of failure by 1%. Patients with larger baseline LA dimension, higher contact force, and post-AF cardiac medications were less likely to fail (OR and 95% CI 0.94 [0.84, 1.05], 0.88 [0.74, 1.05], 0.34 [0.08, 1.38], respectively).

**Table 3:** Multivariate logistic regression for failure of 12-month primary effectiveness endpoint in the EP population (*N* = 78 (the regression model included 72 of the 78 subjects due to 2 subjects missing baseline LA dimension and 4 subjects missing contact force measurement))

Predictors		Odds ratio	95% Wald CI
Gender	Female vs. male	2.98	(0.79, 11.27)
Duration of AF history (months)		1.01	(1.00, 1.02)
Previous AAD: class I**	Yes vs. no	5.32	(1.32, 21.55)
Baseline LA dimension		0.94	(0.84, 1.05)
Average contact force		0.88	(0.74, 1.05)
Post-AF cardiac medications	Yes vs. no	0.34	(0.08, 1.38)

**Predictor statistically significant at 0.05 level

### Safety

From 30-day post-ablation procedure through the 12-month follow-up, there were a total of 15 serious AEs (SAEs) that occurred in 6 patients. All SAEs were considered unrelated to the study device by the investigators. One pericardial effusion, discovered at 92 days post procedure, was classified as serious and related to the procedure. A second pericardial effusion occurred at 180 days post procedure and was classified as possibly procedure related. There were no events of pulmonary vein stenosis and no deaths reported through the effectiveness follow-up period.

## Discussion

This prospective multicenter study has demonstrated safety and efficacy of STSF for the treatment of patients with drug-refractory symptomatic PAF. The Kaplan-Meier estimate of 12-month effectiveness success was 74.9% with a low incidence of SAEs (7.7%) reported between days 30 and 360, the majority (87%) of which were unrelated to the device/procedures.

Compared with traditional 6-hole contact force-sensing catheters, the THERMOCOOL SMARTTOUCH® SF catheter has a 56-hole porous tip and delivers uniform cooling at half the flow rate. This prior study showed that the reduction in flow rate resulted in an overall decrease in fluid delivery of 44.7 to 52.2%, a significant consideration to patients with existing comorbidities, in which fluid overload could adversely affect cardiac function. The positive safety and short-term effectiveness observations in the initial safety phase presented in Chinitz et al. [11] were also associated with improved procedural efficiency with respect to procedure and fluoroscopy times.

Reports of the long-term outcomes of AF ablation with STSF are limited. Previous studies reported 12-month success rates between 80 and 90% in PAF populations (including short-term persistent AF in one study) from single-center or limited multicenter evaluations [12, 13]. Our results represent the experience of a large number of centers (13), therefore likely representative of broader real-world practice. Overall, 12-month effectiveness with STSF was comparable with a previous PAF ablation study (SMART AF) of similar study design using the predecessor 6-hole irrigated CF catheters [8]. Compared with the original THERMOCOOL AF IDE study, conducted almost a decade ago, the current success rates in the SMART AF and SMART SF studies are substantially higher (74–75% vs. 66%) [7]. This finding is further supported by a previous meta-analysis across CF vs non-CF studies showing a decrease in 12-month AF recurrence with the use of CF technology [14–16]. The upward trend in long-term success over time and over CF vs. non-CF ablation studies is encouraging and consistent with the expectation of improved outcomes due to advancement in technologies and catheter ablation experience. Safety of PAF ablation with STSF is further confirmed from long-term safety monitoring of this trial. More recently, a real-world multicenter Italian registry utilizing STSF/Visitag reported higher 12-month success of 90% with lower procedure time (100 min) and fluoroscopy time (6 min) [13].

Compared with those who elected not to be included in the study extension through 12 months of follow-up (non-EP), patients in the EP group tended to have higher percentage of female patients and higher proportion with coronary artery disease. This is reflective of real-world patient decision-making in that patients with symptoms are more likely to seek continue treatment in contrast to patients who feel better who are more likely to exit treatment follow-up. In addition, both gender (being female) and advanced age have previously been shown to be risk factors for recurrence after catheter ablation [17–19]. It is possible that the 12-month success rate observed in the effectiveness evaluation period of this study represented a more symptomatic or sicker patient population than that of the original study, and thus that the success rate in a more representative population would have been higher than what was observed.

This study is limited by the single-arm design and the need for patients to re-consent in order to continue in the effectiveness evaluation phase. Of the 165 patients in the initial safety study, 135 were invited to participate in the effectiveness phase of the study and 80 consented to continue in the study. As such, study participation was not randomized and could have been biased, as observed with some differences in baseline patient characteristics and comorbidities between EP and non-EP patients.

## Conclusion

The SMART SF 12-month evaluation confirms the effectiveness of PAF ablation with STSF. One-year success is high (74.9%) and is coupled with a good long-term safety profile.
